# Strategies of solubility enhancement and perspectives in solubility measurements of pharmaceutical compounds

**DOI:** 10.5599/admet.910

**Published:** 2020-09-27

**Authors:** Christel A.S. Bergström, Antonio Llinas

**Affiliations:** 1 Department of Pharmacy, Uppsala University, Uppsala Biomedical Center, P.O. Box 580, SE-751 23 Uppsala, Sweden; 2 The Swedish Drug Delivery Center, Department of Pharmacy, Uppsala University, Uppsala Biomedical Center, P.O. Box 580, SE-751 23 Uppsala, Sweden; 3 Respiratory, Inflammation and Autoimmunity IMED Biotech Unit, AstraZeneca, Gothenburg, Sweden

The genesis of this theme issue was a conversation we had during the IAPC-8 meeting in Split, Croatia in September 2019. In it, we started the interesting and important discussions surrounding the special session dedicated to ‘State-of-the-art solubility in drug development’. The session was introduced in the preliminary mini-review paper intended to serve as prologue or accompaniment to an upcoming session on solubility at the IAPC-8 meeting in Split, Croatia, 9-11 September 2019 [1]. The session had received a high number of abstracts and hence, was extended from a half-day session to a session spanning over two days with many engaged speakers on topics around solubility, dissolution and enabling formulation strategies. In response to this successful session, Professor Mandic and Professor Tam in their role as editors suggested us to take lead for a special issue dedicated to these topics as they had listened in to the scientific discussions. We were both delighted to take on the role as guest editors. We have been overwhelmed by the response and number of high quality articles submitted for this special issue. Indeed, it was during the course of this work decided to split the special issue into two consecutive issues due to the large number of papers being accepted. With this short editorial, we thank all authors for their excellent contribution to this special issue on solubility enhancement and measurements.

In the first issue of ADMET&DMPK focus is set on computational tools and mathematical modelling useful to understand solubility and dissolution, with Avdeef and Kansy [2] as well as Caron and colleagues [3] focusing their work on compounds in the beyond rule-of-5 (bRo5) chemical space. Avdeef and Kansy explored to what extent computational models established on training sets from the small molecular chemical space were applicable to the bRo5 chemical space. They concluded that Random Forest (RF) regression methods predicted the solubility slightly better for these compounds than the General solubility equation (GSE) and the Abraham Solvation equation (ABSOLV). However, the GSE performed better (for the dataset explored) when the log *P* coefficient was changed from -1 to -0.4, providing less weight to lipophilicity in the predictions. They also concluded that the increased flexibility of the larger molecules in the bRo5 space is one of the underlying reasons for less accurate predictions of traditionally developed models. The latter is also the conclusion from the study by Caron and coworkers [3]. They emphasise the role of flexibility and its importance for chameleonic behaviour, i.e. the ability of the molecule to adopt to the environment it is exposed to. They conclude that the current models are not accurate enough for predictions of this type of compounds and that the solubility models need to be developed for this chemical space. In relation to generation of more predictive solubility models, Mitchell contributes with a paper using three different machine learning models to predict the ‘2019 Solubility challenge dataset’ [4]. It should be noted that this dataset is more traditional, and biased towards the small molecule space, i.e. not the bRo5 space. All three models were based on tree-like classifiers; RF, the Extra Trees algorithm, and a consensus model of the two. It was found the Extra Trees provided the best classifier whereas both RF and the consensus model were slightly better for an explored test set. The author reports RMSEs of 0.95 (tight dataset; Extra Trees) and 1.49 (loose dataset; RF/consensus) indicating the dependency of the chemical space explored. Falcón-Cano *et al*. [5] also explored RF approaches and consensus modelling to predict solubility, with focus on generating better algorithms and improved work-flow during the modelling for data curation and variable selection. In their study the reported RMSE of the generated model is 0.93 log units for the test set taken from the solubility challenge dataset. The authors also contrast their models to other solubility models, providing an updated view on the standing of this field. Improved modelling approaches are further studied by Mecklenfeld and Raabe [6]. They embarked on a study to optimise the force field parameters used for solvation energy calculations. They developed and validated General Amber Force Field (GAFF) parameters by taking into account the Lennard-Jones parameters in combination with implicit polarized charges. The validation was performed by making use of 142 free energies and 100 densities of binary mixtures and resulted in improved models of relative solubilities and fluid phase behaviour. The final two papers in the first special issue focus on mathematical models of dissolution of particles and gastrointestinal motility. Grassi and coworkers [7] described dissolution of poly-dispersed particles in a finite liquid environment, and developed two different equations that take into consideration size reduction, polydispersity and particle geometry (spherical, cylindrical and parallelepided). These mathematical models are useful to, among others, early evaluate the importance of wettability for the dissolution process. Johnson also models dissolution by coupling the dissolution process to the gastrointestinal motility [8]. In this work, the multiple moving plug model was described and used to simulate the effect of the GI motility and particle dissolution on the resulting pharmacokinetic profile of the compound. This work builds on the experimental evidence that the GI fluid exists in water pockets, and hence, the model provides a more physiologically relevant approach to explore the dissolution process in vivo.

In the second issue focus is shifted towards experimental assessment and technologies for profiling and enabling absorption of poorly-water soluble compounds. A series of extensive reviews are provided in this special issue, describing the current standing of electrospun nanofibers for drug delivery and optimised dissolution profiles [9], supercritical fluid technology for controlled production of nano- and microparticles of poorly soluble compounds [10], nanoparticles and their in vivo performance [11] and solubility aspects for proteins [12]. In addition to these timely reviews on important topics for solubility, dissolution and enabling formualations, papers describing original research on assessment and drug delivery of poorly soluble compounds are included. Štukelj *et al*. [13] describes a new methodology to determine dissolution of amorphous powders based on image analysis. In this work, image-based single particle analysis was used to study dissolution of amorphous indomethacin over time and the results were compared to powder XRD analysis. The single particle analysis was useful to study local recrystallization occurring on the particle surface, providing crucial information to understand surface chemistry effects during storage and dissolution. Dissolution was also studied by Gigante *et al*. [14] who set out to perform a global evaluation of the WHO harmonised protocol for equilibrium solubility experiments. The study focused on measurement of 16 compounds for which the biopharmaceutics classification had previously been performed. They successfully validated the current protocol in this extensive collaboration involving 11 laboratories worldwide. The study also indicates that some compounds may have been sorted into the wrong BCS class in previous studies, meriting further exploration. Dissolution evaluation of furosemide products was performed by Medina-Lopéz and coworkers [15]. They studied dissolution for furosemide reference tablets in USP Apparatus 1, 2 and 4, under different experimental conditions. They observed statistically significant differences between USP Apparatus 1 and 2, whereas conditions producing similar dissolution profiles for USP Apparatus 2 and 4 were identified. In the paper authored by Isvoran *et al*. [16] solubility of short oligomers of lactic acid is studied. Polylactic acid has potential to be used in medical applications, and hence the solubility of this polymer is important. The authors conclude that solubility decreases linearly with increased molecular weight and that the short oligomers have limited toxicology but potentially may interact with organic anion transportrs (OATP1B1 and OATP1B3). The final contribution to this special issue is a paper by Prestidge *et al*. [17]. They study a system composed of a lipid-silica hybrid to deliver highly lipophilic antipsychotic compounds. While lurasidone performed as expected in this system, with a 23-fold increase in solubilisation being observed for the developed hybrid system, risperidone instead showed a 2.2-fold reduction when encapsulated in this hybrid. It was shown that differences in ionization between these two compounds resulted in the risperidone adsorbed stronger to the silica than the lurasidone, emphasising the interplay of the drug delivery system and the drug itself for optimal performance.
